# Physicochemical and Quality Properties of Dried Courgette Slices: Impact of Vacuum Impregnation and Drying Methods

**DOI:** 10.3390/molecules26154597

**Published:** 2021-07-29

**Authors:** Magdalena Kręcisz, Bogdan Stępień, Marta Pasławska, Jarosław Popłoński, Kinga Dulak

**Affiliations:** 1 Institute of Agricultural Engineering, Wroclaw University of Environmental and Life Sciences, Chełmońskiego Street 37a, 51-630 Wrocław, Poland; bogdan.stepien@upwr.edu.pl (B.S.); marta.paslawska@upwr.edu.pl (M.P.); 2 Department of Chemistry, Faculty of Biotechnology and Food Science, Wrocław University of Environmental and Life Sciences, 50-375 Wrocław, Poland; jarek.poplonski@upwr.edu.pl (J.P.); kinga.sala@upwr.edu.pl (K.D.)

**Keywords:** vacuum impregnation, courgette, convective drying, vacuum drying, freeze drying, chemical analysis, colour

## Abstract

The aim of this study was to determine the effects that the type of impregnating solution and drying method (freeze drying (FD) and vacuum drying (VD) at 45 °C and convective drying (CD) at 50, 60, and 70 °C) had on the physicochemical and quality properties of courgettes. Courgette slices were vacuum-impregnated (6 kPa) in freshly squeezed onion, kale, and onion and kale (50:50) juices with 3% NaCl solution (N). The application of vacuum impregnation (VI) with impregnating solutions from freshly squeezed onions and kale had a beneficial effect on the bioactive values of courgette. The highest contents of quercetin (41.84 μg/g d.m.) and carotenoids (276.04 μg/g d.m.) were found in courgette impregnated with onion juice after freeze drying. The highest values of lutein and zeaxanthin (216.42 μg/g d.m.) were recorded for courgette impregnated with kale juice and convective dried. By analysing the kinetics of convective drying, the best matching of the logistic model was found. Increasing the drying process temperature from 50 to 70 °C reduced the drying time from 15% to 36%, depending on the type of impregnating solution used. Water activity < 0.6 was recorded for courgette dried by freezing, vacuum, and convection at 60 and 70 °C. Conclusions: The vacuum impregnation process and the impregnation solutions from freshly squeezed vegetables can be used to develop new snacks with high levels of bioactive compounds. The FD method is the most appropriate considering both the bioactive compounds content and the obtained colour and water activity.

## 1. Introduction

The courgette (*Cucurbita pepo* L.) is a popular, seasonally harvested vegetable with a high nutritional value that is grown worldwide. The useful properties of this plant are related to the existence of bioactive compounds, including lutein, β-carotene, and folic acid [1,2]. The courgette plays an important role in a healthy diet because of its high contents of nutrients, including folate (24 μg/100 g), carbohydrates (5 g/100 g), fibre (2 g/100 g), protein, and vitamins and minerals (vitamins C, K, and B6; potassium, 261 mg/100 g; and manganese) [1]. In addition, courgettes do not contain any fat and have a very low energy value (17 kcal per 100 g of product), so it is considered to be a good nutritional addition to a healthy and balanced diet [1,2]. Because of its low energy value and high nutritional value, courgette fruit can be useful in regulating lipid profiles, controlling body weight, and controlling diabetes and cardiovascular diseases. Its consumption is also beneficial for people with eye diseases and pregnant women. This was confirmed by in vitro tests using the HL60 promyelocytic leukaemia cell line and human studies [1]. The courgette belongs to the Cucurbitaceae family, which also includes cucumbers, pumpkins, melons, and watermelons. Courgettes are eaten raw with or without the skin and can also be cooked. Fresh vegetables from the Cucurbitaceae family lose their firmness quickly after cutting and turn brown and rot. Therefore, their consumption period is limited to 1–2 days, after which they become spoiled and lose their beneficial properties. In order to protect vegetables against spoilage, reduce the loss of valuable properties, and allow the possibility of using them out of season, drying is used as one of the methods of food preservation. Thermal treatment aims at inactivating enzymes, reducing water activity, and inhibiting the growth of microorganisms [3,4]. Issues related to the use of pretreatments prior to the drying process to improve the drying properties are of interest to many researchers. Research has shown that the right combination of pretreatment with a specific drying technique can provide a product with the expected properties that can be stored for a long time [5,6].

Quercetin is one of the most valuable naturally occurring compounds in onions, kale, and courgettes in terms of bioactivity. It is a polycyclic aromatic compound of the flavonol group and exhibits antiallergic and anti-inflammatory activity by inhibiting the enzymes involved in the production of leukotrienes and prostaglandins [7,8,9]. It has a strong antioxidant effect, participates in the regulation of blood cholesterol levels, and, together with rutin, affects the strengthening of blood vessels [10].

Increased consumer awareness of health-positive foods accompanies the accelerated development of functional products. Diverse and innovative food preservation methods used in the food industry provide opportunities to improve the functional properties of food products and help to meet market demands [11]. Vacuum impregnation (VI) is a type of osmotic dehydration carried out under vacuum conditions [12]. While the kinetics of osmotic dehydration and the effect of process conditions on product quality are well understood, the vacuum impregnation of biobased materials involves many aspects that remain to be investigated [13]. Vacuum impregnation (VI) is a novel method that uses pressure change to remove intercellular air (with some native fluids) from plant tissue without destroying the initial cellular structure [12,14,15,16,17]. When atmospheric pressure is restored, the pores and intercellular spaces fill with the impregnating liquid, which allows for the direct production of foods with altered quality characteristics [12,14]. VI is widely used to incorporate physiologically active compounds into the porous structures of fruits and vegetables. The application of a vacuum at the beginning of osmotic dehydration increases water loss and the absorption of osmotically soluble components [16,17,18]. The research presented by other authors showed that vacuum impregnation, in which the impregnating solution was calcium or maltose syrup, is therefore recommended as an energy-efficient pretreatment before drying, freezing, or frying various fruits and vegetables [19,20].

Drying is the process of removing water, during which energy and mass transfers take place. This process has been used since ancient times, and the first method applied was solar drying, which is still practised today. Today, several types of dryers are more commonly used, the selection of which has been important in obtaining high-quality products [21]. Hot air drying is a convective drying method. Because of the long drying cycle and high temperature, it can cause degradation of nutrients, flavour, and colour [22]. Reducing the temperature of the convective drying process can positively affect the quality of the dried product. Vacuum drying offers the possibility of lowering the temperature of the drying process because the drying occurs under reduced pressure. VD is an important process, especially for heat-sensitive materials [23]. Dried products obtained by the vacuum method can present higher nutritional value and quality than those obtained by the conventional method [24]. Freeze drying (FD) is one of the best methods for removing water from biological materials, which results in a final product that is of the highest nutritional and sensory quality [23]. The drying process reduces transportation, packaging, and storage costs and provides the opportunity to add value to processed foods [25].

The aim of this study was to investigate the influence of vacuum impregnation conditions, the type of impregnating solutions, and the drying methods (freeze drying, convective drying, and vacuum drying) on the physicochemical properties of dried courgette. For this purpose, the best-fitting mathematical model describing the process of the convective drying of courgettes was selected. Moreover, the determination of bioactive components was carried out for the contents of quercetin, lutein, and zeaxanthin, chlorophylls, and carotenoids, as well as the contents of dry matter, water activity, and colour change.

## 2. Results and Discussion

### 2.1. Dry Matter and Water Activity

Table 1 shows the dry matter content (%) and water activity (-) in all the samples tested. The dry matter content of fresh courgette was 5.68% (corresponding to a moisture content of 94.32%), and similar observations (93.56%) were reported by Eissa et al. [26]. The dry matter content in the dried fruit under study ranged from 75.49% to 96.46%. There was a significant effect of the drying method on the value of the studied parameter (*p*-value = 0.0000). The highest content of dry matter was observed in the samples dried with the freezing method, while the lowest value of the examined parameter was recorded for courgette dried at 50 °C with the convective method. This observation is consistent with the observations of other researchers [26], who obtained dried courgette slices at a temperature of 50 °C and recorded a moisture content of 12.34% (this moisture content corresponded to a dry matter content of 87.66%). This study showed an increase in the value of the examined parameter with an increase in drying temperature during convective drying. The highest average values of the dry matter were obtained from courgette slices subjected to the process of impregnation with the use of kale juice. In the present study, a lower dry matter value was observed for dried courgette subjected to VI using onion juice rather than a NaCl solution. Kowalska et al. [27] reported that by increasing the concentration of sodium chloride, the amount of osmotic substance penetrating the dewatered material increased proportionally. Therefore, the high concentration of sodium chloride causes a high driving force in the osmotic dehydration process, and the dehydration effect and reduction of the water activity of the plant tissue occur rapidly [27]. The use of excessive concentrations, however, may cause adverse changes manifested by damage to cell membranes, which facilitates the penetration of large amounts of substances from the solution into the tissue. During VI there is an exchange of water and mass. The pace of the process slows down until the system reaches a state of equilibrium, which stops the mass transfer process [27]. The difference between the concentration of the solution N (4.2 °Brix) and O (10.5 °Brix) is large; therefore, when transporting a mass of solutions with a higher concentration, more solids are absorbed into the material. Similar observations were reported by other authors when kale leaves underwent impregnation with a 3% NaCl solution or with onion juice combined with 3% NaCl and were subjected to fountain drying [28]. It was found that the type of impregnating liquid had no significant effect on the value of the parameter studied (*p*-value = 0.7015).

The water activity was determined to identify the susceptibility of a product intended for consumption to microbial growth. Chemically pure water has a water activity (aw) of 1. The value of aw decreased with increasing concentrations of the soluble compounds. The lowest water activity at which the development of microorganisms occurred was 0.6 [29]. The sample of fresh courgette had a water activity of 0.988, such a high parameter that all the microorganisms had favourable conditions for development (Table 1). The measurements of the water activity of the dried courgette ranged from 0.218 to 0.670. As in the case of dry matter, the lowest values of the examined parameter were recorded during freeze drying, and the highest values were from convective drying at 50 °C (0.499–0.670). Similar observations were reported by Nawirska-Olszewska et al. [29], who studied ground cherries. Other authors, who subjected Cassia alata to convective drying, observed an increase in water activity values with an increasing temperature for the drying process [30]. This study showed that all the tested samples of dried courgette slices (with the exception of samples convectively dried at 50 °C), namely courgette without impregnation (aw = 0.667), courgette impregnated with onion juice (aw = 0.654), and dried courgette impregnated with kale juice (aw = 0.644), did not require additional protection against the development of microorganisms because of the low value of the parameter studied. The use of different drying methods had a significant effect on the values of the parameter tested (*p*-value = 0.0000). It was observed that as the convective drying temperature increased, the value of the water activity decreased. The application of different impregnating solutions had no effect on the values of the parameter tested (*p*-value = 0.2542). Other authors who dried kale leaves impregnated with 1% NaCl solution and freshly squeezed onion juice with 1% NaCl content did not observe an influence of VI on the values of the parameter tested [28].

### 2.2. Colour Measurement

The results of the measurements of the chromatic coordinates L*, a*, b*; saturation (C*); and total colour change (∆*E*) of the pretreated courgette fruits (VI) with different impregnating liquids and three different drying methods are summarized in Table 2. The values of the chromatic coordinate L* ranged from 42.42 to 87.57, depending on the drying method and impregnating solution used. Both factors had a significant (*p*-value = 0.0000) effect on the chromatic coordinate L*. The highest brightness values were obtained from courgette fruits that were not pretreated. Similar observations were made by other authors during the convective and microwave drying of lemon thyme, in which drying caused a decrease in the L* coordinate value [31]. Other authors observed a decrease in the L* parameter values with an increasing fluidized bed drying temperature [28]. The green colour of kale juice was significantly different from the yellow colour of the courgette flesh, so a decrease in the value of the parameter under study was observed in dried courgette fruit slices with an increasing proportion of kale juice in the impregnating solution. A decrease in the value of the studied parameter was observed with an increase in the convective drying temperature. A similar relationship was shown by Polak R. during the drying of celery leaves in the temperature range of 20–70 °C [32].

Over the course of this study, it was found that the value of the colour discriminant a* was approximately −15.05 to 1.36, depending on the impregnating solutions and drying methods applied (Table 2). The most intense shade of green was recorded for dried courgette that was vacuum impregnated with kale juice, and this was related to the intense green colour of the impregnating solution. Additionally, in this case, the results showed a significant effect of impregnating solutions on the values of the colour discriminant a* (*p*-value = 0.0000). VI resulted in a decrease in the green colour values compared with fresh courgette, except for those obtained by freeze drying. Other authors studying the effect of fluidized bed drying on kale leaves observed an increase in the value of the a* parameter for all the impregnating solutions tested [28]. The highest values of the studied parameter were observed for the samples in which a 3% NaCl solution was used. In the case of convective drying, an increase in the value of the colour discriminant a* was recorded in the CIELab system, which was interpreted as a loss of the green tone. Polak R., while drying celery leaves, showed that the chromatic coordinate a* was in the range of −8.82 to 5.61 throughout the measurement range and observed a loss of the green tone with an increasing drying temperature [32]. At the level of significance α = 0.05, a significant influence of the drying method on the values of the examined parameter was found (*p*-value = 0.0032). In the case of convective drying, a decrease in the green content was observed as the drying process temperature increased.

The values of the colour discriminant b* were positive, which indicated the yellowish hue of the dried courgette slices. The values of the chromaticity coordinate b* were in the range of 24.56–47.91. Both the application of different drying techniques and different impregnating solutions had significant effects on the values of the analysed parameter (*p*-value = 0.0000). The values of the b* colour discriminant decreased with an increasing temperature during convective drying. During convective and vacuum drying, the control samples had lower values of the b* parameter than samples subjected to VI. Similar observations were recorded by other authors while drying pretreated sweet potatoes [33]. Courgette slices dried by the vacuum and freezing methods showed slightly lower values of the examined parameter than samples dried by the convection method. Freeze drying is a method of drying without heating and prevents the degradation of chlorophyll. Other authors who subjected yam slices to freeze and convection drying observed lower values of the b* parameter in the case of freeze drying, which may be due to the loss of the carotenoids responsible for the yellowish hue [33].

The colour change between the dried fruit obtained under different drying conditions and fresh courgette slices was evaluated on the basis of their saturation (C*) and total colour change (∆E). The experimental data revealed that the difference in colour saturation between the dried and fresh raw material increased in all the tested samples, except for the untreated vacuum dried courgette. In this case, a decrease in saturation of 6.52 units was recorded. The decrease in the colour saturation value (C*) in the CIELab system was interpreted as a loss of green and yellow tones, which can be explained by the degradation of chlorophyll a and chlorophyll b during drying [32]. Throughout this study, it was found that the saturation value (C*) decreased with increasing temperatures in the case of convective drying.

**Figure 1:**
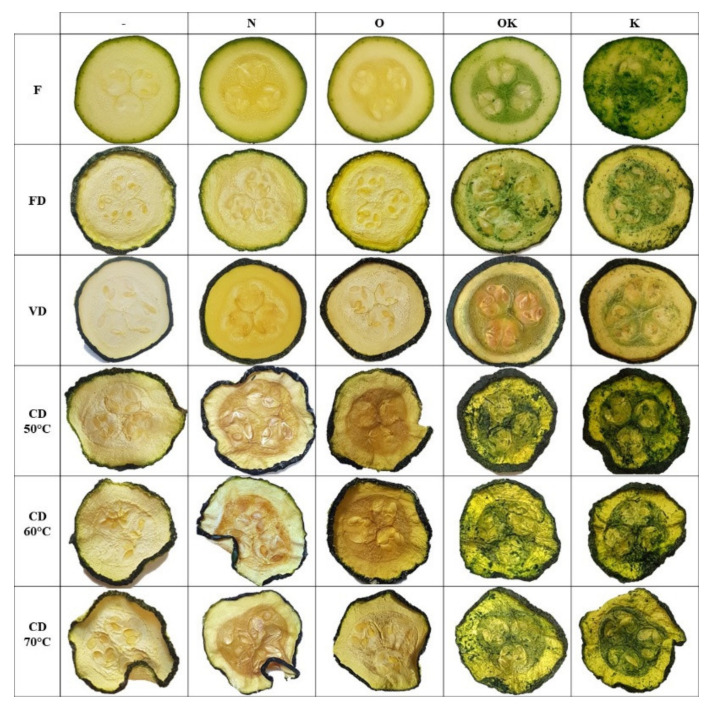
Control sample: fresh courgette (F) dried courgette by freeze drying (FD); vacuum drying (VD); convective drying (CD) at 50 °C (CD 50 °C), 60 °C (CD 60 °C), and 70 °C (CD 70 °C); courgette after VI with 3% NaCl solution (N), onion juice (O), kale juice (K), and onion and kale juice (OK).

The total values of colour (∆E) ranged from 2.82 to 45.90. The closest appearance to the raw material was recorded for courgette samples pretreated with the 3% NaCl solution dried by freeze drying, while the highest values were observed for courgette samples vacuum-impregnated with kale juice and convection dried at 70°C. Smaller colour changes were observed for freeze drying and vacuum drying. The appearance of the samples can be seen in Figure 1. Larger colour differences in courgette were observed for convective drying, in which the highest drying medium temperature was 70 °C. At an air temperature of 50 °C, a decrease in the value of the total colour change was observed, and with a further increase in temperature to 60 °C, an increase in the tested parameter was observed. These results correspond with those of other studies [31,34]. Moreover, Fijalkowska et al. [35] showed that a lower ultrasound frequency resulted in greater colour changes.

### 2.3. Drying Kinetics

On the basis of the experimental data obtained during convective drying and the statistical analysis of the coefficient fitting, it was concluded that water loss in courgette samples is best described by a logistic model. According to other authors, this model is also used to show the effects of drying apples [35]. The statistical coefficients analysed showed the usefulness of the logistic model of the kinetics of courgette slice drying, as evidenced by the high values of R2 (0.9950–0.9996); low root mean square error, RMSE (0.0067–0.0226); low reduced test values, χ^2^ (0.0000–0.0008); and relatively low values of the coefficient of residual variation, Ve (1.4–5.8%) (Table 3). The model parameters a and b are the coefficients of the equation, k is the drying factor; during the CD of courgette slices, the parameters a and b decreased with an increase in temperature up to 60 °C, while parameter k increased. With a further increase in temperature, an increase in all parameters was observed. Other researchers showed values several times higher for the parameters a, b, and k during the convective drying of apple slices at a temperature of 70 °C and a flow rate of 2 m/s [35].

**Figure 2:**
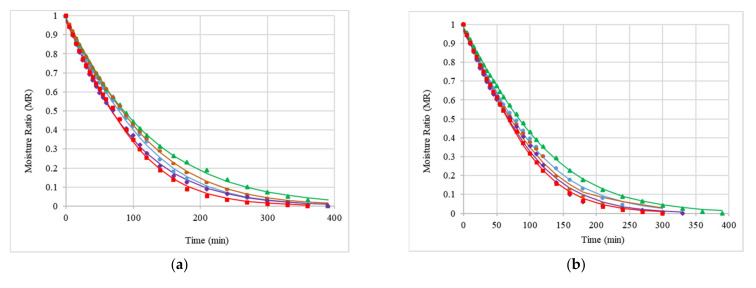

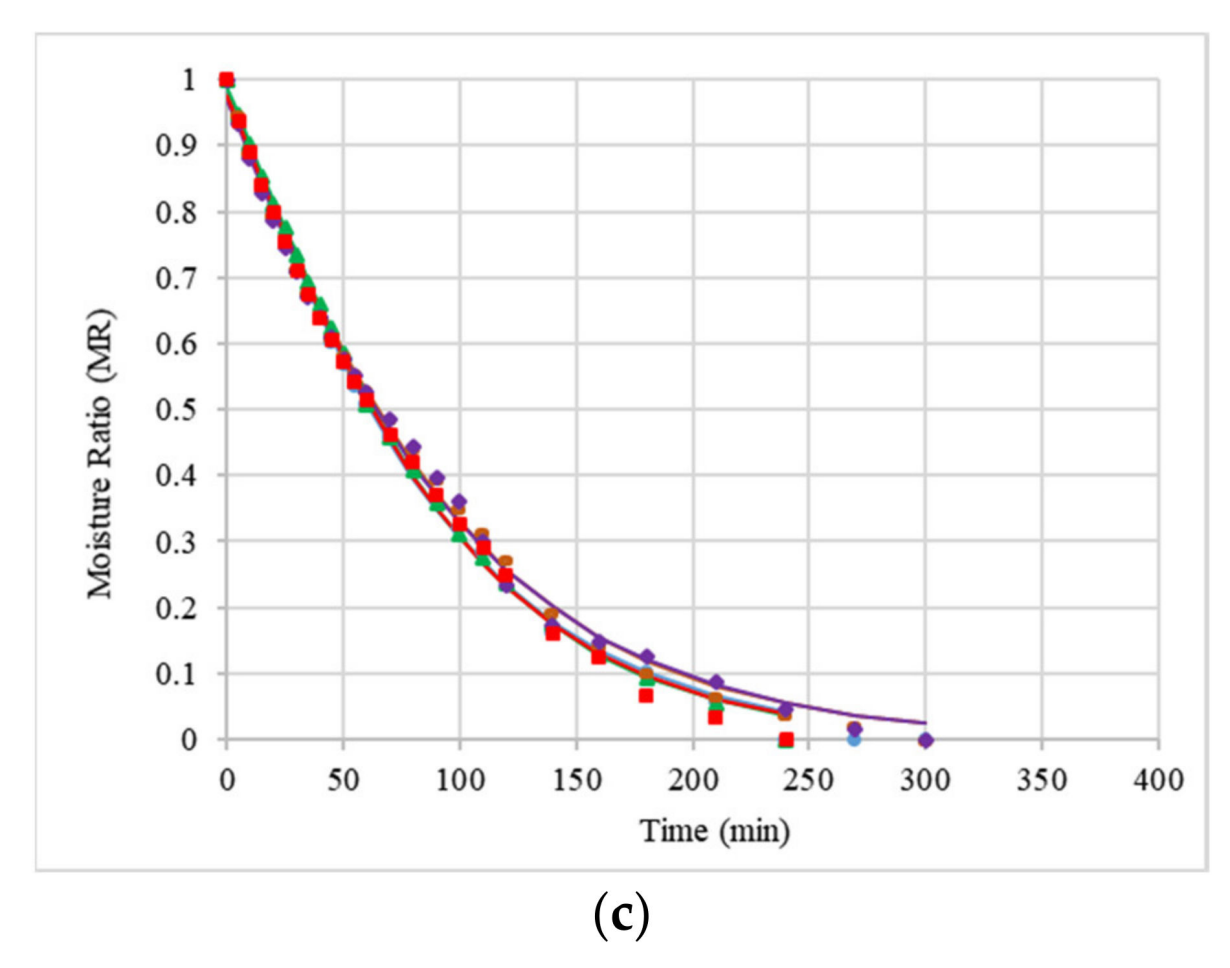
Courgette drying kinetics for convective drying (CD). Control sample: fresh courgette (F) (green), courgette after VI with onion juice (green); courgette after VI with kale juice (blue); courgette after VI with kale (50%) and onion (50%) juice solution (purple); and courgette after VI with NaCl solution (brown). The drying process was conducted at 50 °C (**a**), 60 °C (**b**), and 70 °C (**c**). The parameters of the drying model, R^2^, RMSE, χ^2^, and V_e_, are presented in Table 3.

**Figure 3:**
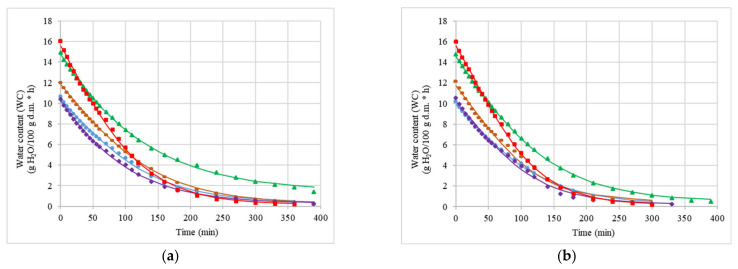

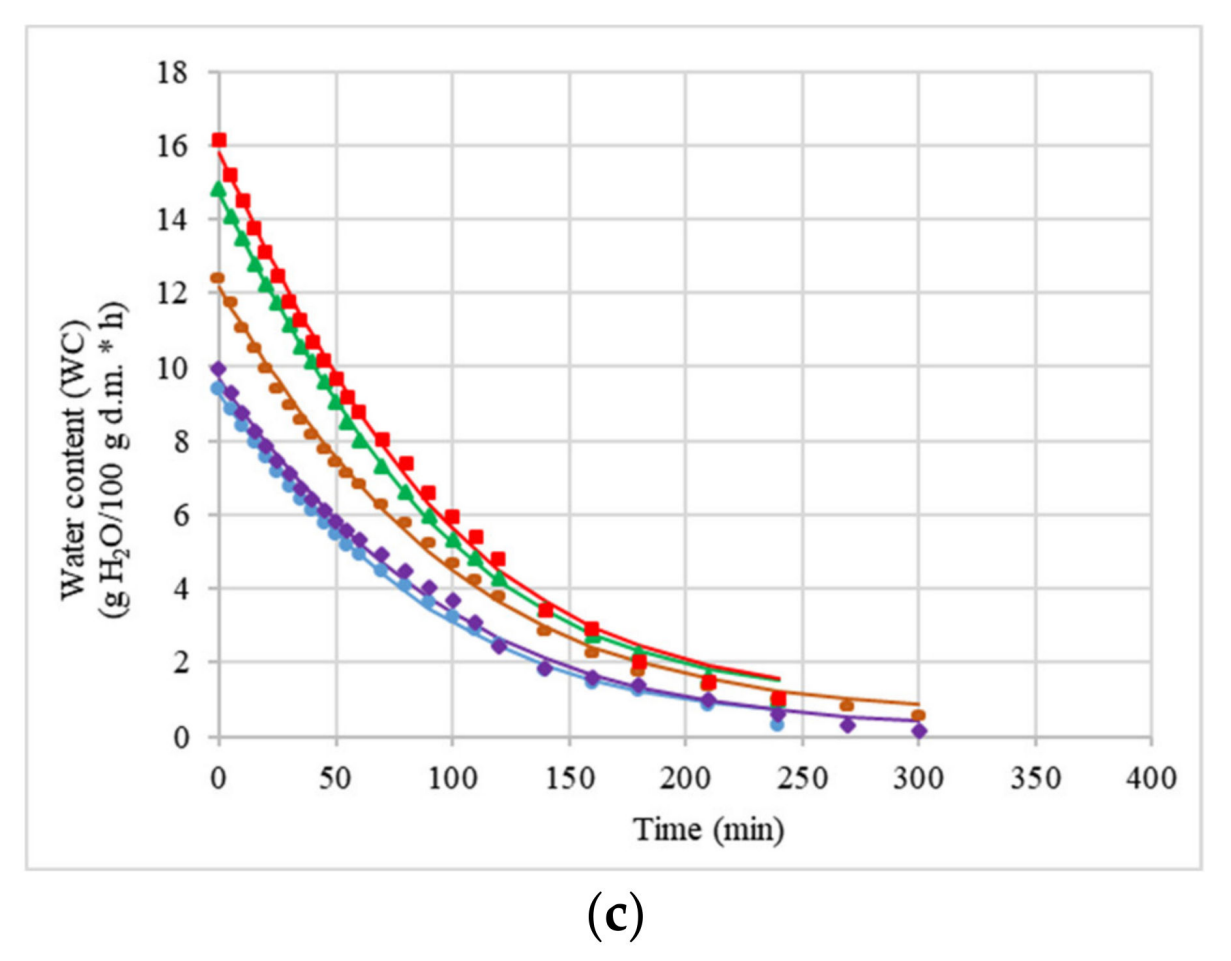
Courgette drying kinetics for convective drying (CD). Control sample: fresh courgette (F) (green), courgette after VI with onion juice (green); courgette after VI with kale juice (blue); courgette after VI with kale (50%) and onion (50%) juice solution (purple); and courgette after VI with NaCl solution (brown). Drying process at 50 °C (**a**), 60 °C (**b**), and 70 °C (**c**).

The drying kinetics of courgette slices subjected to vacuum impregnation in different impregnating solutions and without pretreatment are shown in Figure 2a–c and Figure 3a–c. The process of convective drying was the fastest when the highest temperature of the drying factor (70 °C) was applied and was 270 min for the control sample (F) with the courgette impregnated with O. The application of VI in the case of OK at 50 °C, S at 60 °C, and O at 70 °C did not affect the change in drying time, and in the remaining samples, an increase in drying time was observed. An inverse relationship was observed by Cichowska-Bogusz et al., who subjected apple slices to osmotic dehydration; the osmotic solutions were erythritol, xylitol, and sucrose dissolved in distilled water [36]. The longest drying time was observed for the material impregnated with O, N, and OK solutions during drying at 50 °C. The use of varying temperatures during the drying process reduced the drying time from 60 to 150 min, depending on the impregnating solution used. This finding was similar to the observations of Paslawska et al. made for lemon thyme dried using the CD method [31]. The largest difference in drying times was observed for materials treated with K and O impregnating solutions, and the smallest difference was observed for materials impregnated with S and OK solutions. A rapid decrease and then a gradual decrease in MR values with increasing drying time was observed in the conducted experiments. It was confirmed that the rate of moisture decrease increased with an increasing drying temperature as the heat transfer increased. The decrease in the drying rate may also be due to the increase in drying shrinkage, which caused a decrease in the porosity of the courgette samples. Similar observations were reported by P. Kaushal and H.K. Sharma [37], who investigated the effect of convective drying at 50, 60, and 70 °C of jackfruit (Artocarpus heterophyllus). M. Pasławska et al. [28] investigated the effect of drying temperatures on the drying kinetics for fountain kale. The authors observed an increased drying rate at the initial stage of the process for all the temperatures (70, 90, 110, and 130 °C) and a decrease in the drying rate at the final stages of the process.

The water contents of the courgette samples depended on the impregnating solution used. Figure 3a–c shows the dependence of the applied impregnating liquid as a function of the drying time. The moisture content decreased rapidly and then gradually increased during the drying process. The rate of moisture loss increased with an increasing temperature for the drying process. Similar observations were noted by other authors when jackfruit was subjected to convective drying (50, 60, and 70 °C) [37]. The presented results show that the application of vacuum impregnation resulted in lower water content and longer drying time of courgette slices. The highest water content and the shortest drying time were observed in courgette samples that did not undergo vacuum impregnation. The fresh, intact material has a preserved tissue structure, the pores are not damaged, and there are capillaries that facilitate the transport of water; because of this, the drying process proceeds faster. Depending on the impregnating solution used, the water content of the samples varied. A slightly lower water content compared with that of the control sample was recorded for the material subjected to vacuum impregnation with *O*, and the lowest water contents were recorded for *K* and *OK*. This is probably related to the solid particles contained in the kale juice, which penetrate the material and impede water transport during the drying process.

### 2.4. Chemical Properties

The effect of impregnation treatment on the contents of the selected bioactive compounds in courgette slices was found to be negative or positive depending on the type of impregnant solution used. Impregnation in a salt solution (N) caused the elution of valuable components from the material, whereas the impregnation of courgette slices in onion juice and kale juice led to a significant increase in the contents of selected bioactive compounds obtained. This resulted from the introduction of valuable compounds from the vegetables from which the juice was squeezed. Impregnation in a salt solution is a typical osmotic dehydration process that causes an intensive outflow of internal tissue water together with native bio components [38], whereas vacuum impregnation in vegetable juices let to incorporate valuable ingredients from the juice into the material processed [12].

The impregnation of courgette in an aqueous solution of table salt resulted in the complete elution of quercetin, and a partial elution resulted if the impregnation was in vegetable juice with salt. Impregnating courgette in onion and kale juices, which come from vegetables rich in quercetin, led to a significant increase in the quercetin content observed in the samples, from 1.82 μg/g d.m. in the fresh material to 51.00 μg/g d.m. in slices impregnated in onion juice and 11.00 μg/g d.m. in slices impregnated in onion and kale juice (Table 4).

Compounds from the group of chlorophylls and carotenoids are equally important components of the human diet [39,40] mainly because of their strong antioxidant properties, protection against many diseases of civilization, and ability to delay the effects of ageing. The levels of carotenoids and chlorophylls after impregnation and after drying in different conditions were compared with fresh courgette.

The chlorophyll content changed after impregnation depending on whether the impregnant contained compounds from this group or not. The greatest decrease in the chlorophyll content was recorded after impregnation in an aqueous salt solution (33%), and there was a smaller decrease using onion juice (12%), while after impregnation in kale juice, there was a significant increase (by 41% for kale and onion juice and by 52% for kale juice). In the previous experiments, when kale leaves were impregnated with an onion juice or a salt solution [28], an outflow of chlorophyll was also observed, but there was not a large difference between the salt solution and onion juice (chlorophyll dropped by 11 and 12%, respectively). Impregnation also affected the carotenoid content of courgette slices. The application of an aqueous salt solution and onion juice caused decreases in the content of carotenoids by 6.5% and 3.5%, respectively, while after impregnation in kale juice with and without onion juice, an increase in the content of compounds from this group of 10–17% was observed. The results were in agreement with those obtained for kale leaves [28], in which a 22% loss of carotenoids was noted after impregnation in the salt solution, whereas after impregnation in onion juice, no significant loss of carotenoids was noted. We also analysed the contents of two extremely important compounds from the carotenoid group—lutein and zeaxanthin—which are important for the proper functioning of the macula in the eye [41]. These compounds were determined together, and before and after the impregnation, and only impregnation in the aqueous saline solution contributed to their elution (loss of 10%), whereas the vegetable juices introduced these compounds into the courgette tissue, which resulted in increases of approximately 2% in the case of onion juice and approximately 18% for kale juice.

On the basis of the analysis of the effects of the impregnation of courgette slices with selected solutions on the contents of selected compounds with health-promoting properties, it was found that the solution of table salt adversely affected the healthiness of the studied samples by eluting valuable bioactive compounds, while impregnation in vegetable juices rich in compounds that are valuable for health significantly increased the nutritional value of the courgette.

Drying caused a decrease in the bioactivity of courgette, both impregnated and non-impregnated, but there was no clear effect of the method used on the degree of degradation of the bioactive compounds studied.

The loss of quercetin content for all the applied drying methods was recorded; the lowest was a loss of 4.2% when drying was carried out by the vacuum method after prior impregnation in onion and kale juice, and the highest loss was when drying by convection at 50 °C after prior impregnation in different solutions (at 73–100%). A beneficial effect of increasing the convective drying temperature in the range of 50–70 °C for the preservation of quercetin was found—drying at 50 °C led to a significant or complete breakdown of quercetin in all the experiments, while increasing the temperature and at the same time shortening the dehydration time enabled the preservation of this compound at high levels. Finally, the highest quercetin content in the material dried by all the techniques was determined in the courgette impregnated with onion juice and subjected to convective drying at 70 °C: 49.45 μg/g d.m.

The drying of courgette slices using the selected methods resulted in a decrease in the contents of compounds from the chlorophyll group; the greatest was a 71% decrease (in relation to the level after impregnation in kale and onion juice) after the application of convective drying at 70 °C, and the lowest was a decrease of 3% after the application of freeze drying after prior impregnation in onion juice. Because of the high degree of chlorophyll preservation, freeze drying proved to be the least destructive technique for drying courgette. The obtained results for zucchini are in line with the results for kale leaves dried by fluidization, in which a drop in the chlorophyll content was observed, lowest at a temperature of 90 °C [28]. On the contrary, a different trend was observed by Eissa et al. [26], as they noticed an increase of chlorophyll content in zucchini dried by solar (by 32%) and oven drying methods (by 104%).

Drying also proved to be destructive to the carotenoids present in the material; the most evident loss occurred when convection drying at 50 °C was used (58–90% loss of carotenoid content, depending on the impregnant used). It was not clear which drying technique was the best, as the results also depended on the impregnant used. Additionally, when drying slices without freeze drying, there was a 10% decrease in the carotenoid content, whereas for convection drying at 60 °C of slices impregnated in kale juice, the decrease in the carotenoid content was only 1%. The most conservative method of drying courgette that was not impregnated regarding the levels of lutein and zeaxanthin was freeze drying. This resulted in a decrease in the contents of these compounds of approximately 2%. The content of lutein and zeaxanthin in fresh green zucchini was 184.91 μg/g d.m.; other researchers reported a lutein content of 1036.9 μg/g d.m. for yellow courgette and 135 μg/g d.m. for light green courgette *Epikarp* [42]. The positive effect of freeze drying on the total carotenoid content was also reported by López-Lluch et al. [34] in loquat fruit dried by different methods, but the authors noticed a much higher content of zeaxanthin in material that was convectively dried rather than freeze dried. As carotenoids are destroyed by heat treatment, the decrease in the total carotenoids was expected after drying zucchini, but the effect of rising temperature was positive on carotenoid content, not negative. This trend is in agreement with other authors, Azevedo-Meleiro et al. [43], Samia [44], and Farina et al. [45], who also indicated an improvement in carotenoid retention by reducing the processing time caused by rising the temperature. In the case of drying courgette impregnated in kale juice, a loss of 1% was observed, while the other impregnants did not protect these compounds from drying-induced destruction. In courgette impregnated in onion juice, a greater loss of lutein and zeaxanthin was observed after drying, and the most destructive drying method in all the variants of the experiment was convective drying at 50 °C (loss of 47–93%).

With regard to the degree of degradation of valuable nutrients in the analysed material, convective drying at 50 °C proved to be the least favourable drying method, while freeze drying and convective drying at 70 °C proved to be the most favourable.

## 3. Materials and Methods

### 3.1. Material Preparation

Fresh courgette with an initial moisture content of 94.32% was purchased from a local vegetable market (commercial products from Lower Silesia, Poland) and stored at 4 ± 2 °C in a refrigerator prior to testing in order to reduce respiration and biological and chemical changes. Immediately before testing, the courgette was washed, blotted dry, and cut into 2 ± 0.1 mm-thick slices.

### 3.2. Impregnating Solutions

Different impregnation solutions were used in the vacuum impregnation process, as shown in Table 5. The juices were obtained using a juicer (AE 3532 CTC Clatronic, Warsaw, Poland). The refractive index was measured using an Atago Digital Brix Refractometer PAL-3 (Atago Co., Ltd., Tokyo, Japan).

### 3.3. Vacuum Impregnation

Vacuum impregnation was performed in a prototype plant located at the Institute of Agricultural Engineering, Wroclaw University of Life Sciences (Wroclaw, Poland) [12]. Samples weighing 120 g were placed in a perforated stainless steel vessel. The vessel was placed in the impregnation chamber and was subjected to a reduced pressure maintained at 0.06 MPa for 2 min at 22 °C. Then, 700 g of the impregnation solution was added, and the test material was kept in it for 4 min. After this time, atmospheric pressure was restored, and the next stage of the impregnation was carried out for 20 min. The vacuum impregnation process was carried out according to Pasławska et al. [28] with our own modifications. Next, the impregnating solution was drained from the courgette, which was slightly dried with filter paper, weighed with an accuracy of ± 0.001 g, and subjected to the drying process.

### 3.4. Drying Methods

The courgette slices were dried using a convective dryer (CD) designed and built at the Institute of Agricultural Engineering (Wroclaw, Poland), which has been described in many publications [46]. The convective dryer, equipped with six chimneys, can dry 6 material samples simultaneously. The drying medium was hot air with temperatures of 50, 60, and 70 °C at a flow rate of 0.5 m·s^−1^. Samples of 50 g were placed in a single layer in baskets. The measurement was carried out in two repetitions; the average of the measurements was taken as the result. During drying, the weight loss was recorded (Axis A5000, Radwag, Radom, Poland) as follows: every 5 min for the first hour, every 10 min for the second hour, every 20 min for the third hour, and then every half hour.

Vacuum drying (VD) was carried out in a laboratory dryer, V0101 (Memmert, Schwabach, Germany), at 45 °C with varying pressures from 10 kPa for 30 h. The conditions were determined by preliminary tests.

Freeze drying (FD) was performed in an installation (Free-Zone 4.5 L, Labconco, Fort Scott, KS, USA) at −50 °C at a constant pressure of 5 Pa for 24 h. All the shelves reached a temperature of 22 °C. The prepared courgette slices were individually spread on a tray, and the vegetables were frozen at − 18°C for 48 h at a cooling rate of 1 °C·min^−1^ in a home freezer.

Dried food was vacuum packed (PA/PE clear film, 80 μm thick, with maximum permeability of 6 g·m^−1^ 24 h for water vapour and <40 cm^−3^·m^−1^·24 h for oxygen) and stored in the absence of light at room temperature, followed by the scheduled tests.

### 3.5. Mathematical Modeling

To describe the kinetics of the convective drying of courgette slices, the relative water content was calculated as follows:

(1) $$MR=\frac{M_{t}-M_{r}}{M_{0}-u_{r}}$$

where *M*_0_ is the initial water content (g water/g dry matter), *M*_t_ is the water content after time t (water/g dry matter), and Me is the equilibrium water content (g water/g dry matter) [37].

For the mathematical description of the drying curves for the courgette slices, the models most commonly used in the literature were selected, as shown in Table 6. Regression analysis of the drying curves was performed using Microsoft Excel 2013.

In order to select the appropriate model that best described the process flow, an *RMSE* analysis was performed, along with aiming to reduce the *X^2^* test values and coefficient of variation of the residual *V_e_*, using the relationship:

(2) $$R^{2}=\frac{\sum_{i=1}^{N}{\left(MR_{i,p}-MR_{p}\right)}^{2}}{\sum_{i=1}^{N}{\left(MR_{i,e}-MR_{p}\right)}^{2}},$$

(3) $$RMSE=\sqrt{\frac{\sum_{i=1}^{N}{\left(MR_{i,p}-MR_{i,e}\right)}^{2}}{N}}$$

(4) $$\chi^{2}=\frac{\sum_{i=1}^{N}{\left(MR_{i,p}-MR_{i,e}\right)}^{2}}{N-n}$$

(5) $$V_{e}=\frac{\sqrt{\chi^{2}}}{Y}\cdot 100\%,\;$$

where *MR_i,p_* is the calculated (predicted) relative water content, *MR_i,e_* is the experimental relative water content, *N* is the number of observations, *n* is the number of parameters in the model equation, and *Y* is the mean experimental relative water content, *MR* [12].

The high values of *R^2^* and the lowest values of *χ*^2^ and *RMSE* showed that the model described the experimental data obtained well.

### 3.6. Dry Matter and Water Activity

A vacuum dryer, V0101 (Memmert, Schwabach, Germany), was used to determine the dry weights of the fresh and dried courgette slices. The courgette samples (0.5 g) were accurately measured using an electronic balance (AS160/C/2, Radwag, Radom, Poland; accuracy of measurement: ± 0.0001 g) and dried at 70 °C under reduced pressure (3 kPa) until a constant weight was obtained. The measurements were performed three times. The average of the three repetitions was taken as the result and expressed as a percentage [53].

The water activity in fresh and dried material was determined using the AquaLab 4TE ± 0.003 apparatus (AquaLab, Warsaw, Poland) according to the manufacturer’s instructions, and the temperature was constant (25 °C). The results are based on four replicates.

### 3.7. Colour Measurement

The colour measurement of fresh and dried courgette enriched with the impregnating solutions was performed using a Minolta Chroma Meter CR-200 colourimeter (Minolta Corp., Osaka, Japan). The surface colour of the test samples was measured. The CIE-Lab scale was used to evaluate L* for brightness, a* for (+) redness/(−) greenness, and b* for (+) yellowing/(−) blueness, respectively. A D65 light source and a standard colourimetric observer with a field of view of 10° were used. A colour measurement was performed using a colourimeter with a measuring aperture 0.008 m in diameter. The measuring window was placed at a distance of 0.010 m from the sample.

The total colour change (∆*E*) between dried (L*_sample_, a*_sample_, and b*_sample_) and fresh courgette fruit (L*_control_, a*_control_, and b*_control_) was calculated according to the equation [54]:

(6) $$∆E=\sqrt{\left[{\left(L_{sample}^{*}-L_{control}^{*}\right)}^{2}+{\left(a_{sample}^{*}-a_{control}^{*}\right)}^{2}+{\left(b_{sample}^{*}-b_{control}^{*}\right)}^{2}\right]}$$

The colour saturation (C*), used to determine the degree of difference in hue, as compared to a grey colour of the same brightness, was considered to be a quantitative attribute of colour. Higher saturation values are associated with higher colour intensities for dried courgette fruit, as perceived by humans. The colour saturation was calculated according to the equation [55]:

(7) $$C^{*}=\sqrt{a^{*2}+b^{*2}}$$

### 3.8. Chemical Properties

#### 3.8.1. Substances and Sample Preparation

The compounds quercetin, lutein, and zeaxanthin used in the analytical studies were purchased from Extrasynthese (France) and were of analytical grade. The samples of courgette were treated according to the drying section and were sliced and mortar ground. Then, approximately 100–300 mg of each sample was transferred to a glass vial (10 mL), and 5 mL of extraction solvent was added; the vial was then closed tightly, and the sample was sonicated in a water bath at 40 °C for 15 min (Bransonic^®^ cpx). Five consecutive extraction solvents were used: (1) an ethyl acetate:hexane mixture (1:1 *v*/*v*), (2) ethyl acetate, (3) an ethyl acetate:methanol mixture (1:1, *v*/*v*), and (4 and 5) methanol; combined organic extracts were evaporated on a rotary evaporator to dryness, and the samples were dissolved in 2 mL of methanol, aided by sonication, and centrifuged for 1 min at 20,000 rpm (Eppendorf Centrifuge 5420). Then, 100 µL of the supernatant was transferred to an amber vial with 900 µL of methanol, which was closed and stored at −80 ℃ for analysis, and the rest of the supernatant (about 1.5 mL) was stored in an Eppendorf vial (2.0 mL) at –80 ℃ for further analysis. All the samples were prepared in triplicate.

#### 3.8.2. Evaluation of Total Carotenoids and Total Chlorophyll

The total carotenoid and total chlorophyll contents were evaluated using the method described by Wellburn [56]. Methanol samples stored in Eppendorf tubes were incubated at 20 °C for 10 min in a water bath; then, each sample was transferred to a quartz cuvette and analysed at 470, 552, and 665 nm using a UV5Nano (Mettler Toledo, Zurich, Switzerland). If the AU at any wavelength was higher than 1.2, the samples were diluted with methanol to fit within 0.2–0.8 mAU. The total carotenoid and total chlorophyll contents were determined using the equations presented by Wellburn [56], including the dilutions, if applicable, and the dry weight masses of the courgette samples.

#### 3.8.3. Evaluation of Quercetin and Combined Lutein/Zeaxanthin Contents in Courgette

The quercetin and combined lutein/zeaxanthin contents were evaluated using HPLC-MS (Shimadzu Prominence-I LC-2030C 3D Plus equipped with a triple quadrupole MS detector, Shimadzu LCMS-8045) operated in ESI-MS mode with the following parameters: needle potential, 4.5 kV; nebulizing gas flow, 3 L/min; heating and drying gas flow rate, 10 L/min; interface temperature, 300 °C; and heat block temperature, 200 °C. Nitrogen was used as the source gas, and argon was used as a collision gas (230 kPa).

For all the standards, ionisation optimisation was performed to evaluate the precursor ion, product ion, collision energy (CE), and quadrupole voltages. The parameters obtained were further applied for quantitation in a multi-reaction monitoring (MRM) analysis of parent ion→product ion transitions: 568.6→476.35 (CE: −17.0) and 568.6→338.3 (CE: −20.0) for lutein and zeaxanthin (as both shared the same parameters), and 301.2→151.25 (CE: 20.0) and 301.2→179.25 (CE: 17.0) for quercetin.

Chromatographic separations were achieved with a Kinetex C18 2.6 µm (3 × 100 mm) analytical column at 40 °C using a 0.1% formic acid water solution (solvent A) and 0.1% formic acid acetonitrile solution (solvent B) gradient at a 0.45 mL/min flow. The elution gradient was as follows: initial condition, 15% solvent B; 0→3 min, 15% solvent B; 3→10 min, 100% solvent B; 10→15 min, 100% solvent B; 15→17.5 min, 15% solvent B; and 17.5→21 min, 15% solvent B. Prior to and after every injection, the needle and injection loop were purged 5 times with a 0.1% formic acid acetonitrile/water (1:1 *v*/*v*) solution.

For each of the measured compounds, a 10-point calibration curve was prepared in the ranges of 100 to 0.5 ng/mL for quercetin and 210 to 0.21 ng/mL for lutein by preparing serial dilutions in MeOH (triplicate). The mean values obtained were plotted on a graph of ion count*min versus concentration and showed coefficients of determination (R^2^) of 0.997 and 0.998, respectively, with calculated limits of quantification (LOQs) of 0.24 µg/g d.w. for quercetin and 0.12 µg/g d.w. for the lutein/zeaxanthin content.

### 3.9. Statistical Analysis

Statistical analysis based on two-factor analysis of variance (ANOVA) was performed using Statistica 13 software (Statistica, Tulsa, OK, USA). The results are presented as means ± standard deviation. Tukey’s test was used to evaluate the significance of differences (*p* ≤ 0.05) between the mean values. A mathematical description was performed using Microsoft Excel 2013.

## 4. Conclusions

This study investigated the effects of different drying methods and different impregnating liquids on the physicochemical and quality properties of courgette. Both the drying method and the type of impregnating liquid had significant effects on the changes in the drying effects and nutritional values of the dried products. The application of different drying methods had a significant effect on the water activity; dry weight; chromatic coordinates L*, a* and b*; and bioactive properties of courgette. The products obtained by freezing, vacuum, and convective drying at 60 and 70 °C had the microbiological properties required to prevent spoilage. Drying caused a decrease in the value of the L* parameter in all samples. Increasing the drying temperature during the convection process caused a decrease in green (a*) and yellow (b*) colour content. The smallest colour differences between fresh and dried courgette were observed for courgette impregnated and dried using sublimation and for courgette not subjected to IV, and the largest was for courgette impregnated with K for all the drying methods tested. The use of different impregnating solutions had a significant effect on the values of the parameters L*, a*, b*, quercetin, lutein and zeaxanthin, carotenoids, and chlorophylls. The drying kinetics were best described by our logistic model. An increase in the drying temperature resulted in a reduction in the duration of the process, from 15% to 36%, depending on the type of impregnating liquid used.

In conclusion, the best method considering bioactive components content, dry matter, and water activity value was FD. Drying kinetics showed that the use of CD 70 significantly reduced the required time of drying. The application of vacuum impregnation extended the drying time. The colour of the material after VI and drying was found to be changed to the colour of kale juice, which shows that VI can be used for natural colouring. In summary, a new type of dried vegetable snack supplemented with the addition of freshly squeezed juices could be a high-quality, nutritionally valuable snack that replaces traditional crisps. In addition, the use of vacuum impregnation and other impregnating solutions can be an inspiration for the design of new functional food products.

## 5. Patents

Patent Poland, no 421913. Vacuum impregnating machine and method for initial processing of material. Wrocław University of Environmental and Life Sciences, Wrocław, PL. Authors: Bogdan Stępień, Radosław Maślankowski, Leszek Rydzak, and Marta Pasławska.

## Figures and Tables

**Table 1:** Dry matter and water activity in dried courgette samples. Means within one column with a different superscript letter are significantly different homogeneous groups (*p* < 0.05).

Drying Method	Material	Dry Matter [%]	Water Activity [-]
CD 50	F	84.92 ± 0.72 ^d^	0.670 ± 0.037 ^n^
	N	80.37 ± 0.32 ^b^	0.499 ± 0.008 ^k^
	O	75.49 ± 0.33 ^a^	0.654 ± 0.039 ^m,n^
	K	81.53 ± 0.89 ^c^	0.644 ± 0.030 ^m^
	OK	80.86 ± 0.61 ^b^	0.556 ± 0.024 ^l^
CD 60	F	90.92 ± 0.59 ^e^	0.349 ± 0.007 ^g,h,i^
	N	94.76 ± 0.80 ^i,j^	0.355 ± 0.010 ^g,h,i^
	O	91.84 ± 0.28 ^f^	0.402 ± 0.006 ^j^
	K	90.50 ± 0.90 ^e^	0.425 ± 0.005 ^j^
	OK	93.56 ± 0.37 ^h^	0.369 ± 0.006 ^h,i^
CD 70	F	91.68 ± 0.35 ^f^	0.276 ± 0.006 ^c,d^
	N	96.76 ± 0.11 ^k,l^	0.297 ± 0.006 ^d,e^
	O	92.82 ± 0.18 ^g^	0.335 ± 0.013 ^f,g^
	K	94.38 ± 0.31 ^i^	0.303 ± 0.008 ^e^
	OK	96.10 ± 0.20 ^k^	0.330 ± 0.016 ^f,g^
VD	F	94.65 ± 0.12 ^i,j^	0.318 ± 0.006 ^e,f^
	N	95.23 ± 0.04 ^j^	0.340 ± 0.009 ^f,g^
	O	96.46 ± 0.16 ^k^	0.348 ± 0.013 ^g,h^
	K	96.10 ± 0.05 ^k^	0.317 ± 0.003 ^e,f^
	OK	96.33 ± 0.01 ^k^	0.375 ± 0.002 ^i^
FD	F	97.08 ± 0.00 ^l,m^	0.218 ± 0.006 ^a^
	N	95.57 ± 0.17 ^m,n^	0.255 ± 0.044 ^b,c^
	O	98.12 ± 0.11 ^n,o^	0.222 ± 0.004 ^a^
	K	97.68 ± 0.07 ^m,n^	0.239 ± 0.003 ^a,b^
	OK	98.47 ± 0.18 ^o^	0.255 ± 0.009 ^b,c^

CD: convective drying (50 °C, 60 °C, and 70 °C), VD: vacuum drying, FD: freeze drying, F: courgette without pretreatment, N: 3% NaCl solution, O: onion juice + 3% NaCl, K: kale juice + 3% NaCl, and OK: onion and kale juice (50:50) + 3% NaCl.

**Table 2:** The colour parameters L*, a*, b*, C*, and ΔE (total colour difference) in dried courgette samples. Means within one column with a different letter superscript are significantly different homogeneous groups (*p* < 0.05).

Drying Method	Material	L*	a*	b*	C*	Δ*E*
-	F	87.57 ± 0.82 ^p^	−3.28 ± 0.11 ^k,l^	30.87 ± 0.67 ^b^	31.04	-
CD 50	F	84.94 ± 0.71 ^n,o^	−4.76 ± 0.05 ^q^	36.95 ± 0.51 ^h,i,j^	37.96	6.79
	N	77.52 ± 0.82 ^k^	−2.92 ± 0.04 ^m,n^	43.95 ± 0.46 ^o^	44.05	16.50
	O	60.53 ± 0.57 ^e,f^	−2.69 ± 0.05 ^o^	38.33 ± 1.01 ^k,l^	38.42	28.60
	K	52.48 ± 0.48 ^c^	−15.05 ± 0.32 ^a^	41.57 ± 0.68 ^n^	44.21	38.53
	OK	62.07 ± 0.70 ^f^	−12.01 ± 0.31 ^c^	42.88 ± 0.52 ^o^	44.53	29.51
CD 60	F	83.24 ± 1.30 ^m,n^	−3.80 ± 0.08 ^j^	34.74 ± 0.68 ^f,g^	34.95	5.83
	N	67.61 ±0,47 ^h^	−2.83 ± 0.05 ^n,o^	38.01 ± 1.01 ^j,k,l^	38.12	21.20
	O	60.19 ± 0.36 ^e,f^	−2.32 ± 0.04 ^i^	36.45 ± 0.33 ^h,i^	36.47	28.02
	K	48.57 ± 0.64 ^b^	−13.31 ± 0.60 ^b^	39.09 ± 0.67 ^l^	41.29	41.10
	OK	60.79 ± 1.03 ^e,f^	−10.55 ± 0.49 ^e^	40.89 ± 0.74 ^m,n^	42.23	29.50
CD 70	F	77.47 ± 0.69 ^k^	−3.14 ± 0.06 ^l,m^	31.69 ± 0.48 ^b,c^	31.85	10.13
	N	61.77 ± 0.59 ^f^	−2.35 ± 0.03 ^o^	35.90 ± 0.24 ^g,h^	35.98	26.30
	O	59.37 ± 1.09 ^e^	−1.25 ± 0.03 ^p^	34.16 ± 0.67 ^e,f^	34.18	28.46
	K	42.42 ± 1.45 ^a^	−11.47 ± 0.52 ^d^	31.89 ± 0.53 ^c,d^	33.89	45.90
	OK	55.51 ± 1.20 ^d^	−8.65 ± 0.47 ^g^	38.12 ± 0.42 ^j,k,l^	39.09	33.31
VD	F	75.89 ± 0.53 ^k^	−1.58 ± 0.14 ^p^	24.56 ± 0.82 ^a^	24.61	13.38
	N	71.35 ± 1.00 ^i^	1.36 ± 0.09 ^r^	30.68 ± 0.40 ^b^	30.71	13.87
	O	81.30 ± 0.50 ^l,m^	−1.34 ± 0.06 ^p^	32.42 ± 0.40 ^c,d^	32.45	6.74
	K	71.37 ± 2.51 ^j^	−6.61 ± 0.25 ^h^	39.08 ± 0.70 ^l^	39.64	18.46
	OK	76.95 ± 1.08 ^k^	−3.55 ± 0.08 ^j,k,l^	37.64 ± 0.64 ^i,j,k^	37.81	12.60
FD	F	80.83 ± 1.06 ^l^	−4.45 ± 0.28 ^i^	33.40 ± 1.60 ^d,e^	33.70	7.29
	N	85.73 ± 0.82 ^o,p^	−4.70 ± 0.05 ^i^	32.47 ± 0.74 ^c,d^	32.81	2.82
	O	75.85 ± 0.70 ^k^	−3.63 ± 0.04 ^j,k^	34.44 ± 0.54 ^e,f^	34.63	12.26
	K	59.01 ± 1.60 ^e^	−13.01 ± 0.38 ^b^	40.32 ± 1.38 ^m^	42.37	31.62
	OK	65.32 ± 4.60 ^g^	−9.85 ± 0.44 ^f^	38.92 ± 0.59 ^l^	40.15	24.56

CD: convective drying (50, 60, and 70 °C), VD: vacuum drying, FD: freeze drying, F: courgette without pretreatment, N: 3% NaCl solution, O: onion juice + 3% NaCl, K: kale juice + 3% NaCl, and OK: onion and kale juice (50:50) + 3% NaCl.

**Table 3:** Parameter values a, b, k, R^2^, RMSE, χ^2^, and V_e_ of the function describe the drying kinetics for courgette samples dried by convection at different temperatures.

Drying Method	Material	a	b	k	k_i_	c	*R^2^*	RMSE	χ^2^	V_e_ (%)
**Page Model**										
CD 50	F	1.1427	-	0.0057	-	-	0.9965	0.0198	0.0004	4.4
	N	1.1181	-	0.0050	-	-	0.9989	0.0116	0.0001	2.5
	O	1.0274	-	0.0072	-	-	0.9990	0.0099	0.0001	2.1
	K	1.1216	-	0.0053	-	-	0.9969	0.0186	0.0003	4
	OK	1.0418	-	0.0087	-	-	0.9985	0.0125	0.0002	2.9
CD 60	F	1.0631	-	0.008	-	-	0.9967	0.0270	0.0008	5.6
	N	1.1316	-	0.0058	-	-	0.9932	0.0027	0.0008	5.9
	O	1.1181	-	0.0050	-	-	0.9989	0.0116	0.0001	2.5
	K	1.0631	-	0.0080	-	-	0.9929	0.0297	0.0010	6.1
	OK	1.0631	-	0.0080	-	-	0.9945	0.0298	0.0010	6.7
CD 70	F	1.0873	-	0.0080	-	-	0.9955	0.0204	0.0005	4.3
	N	1.0435	-	0.0091	-	-	0.9966	0.0182	0.0004	4
	O	1.1335	-	0.0065	-	-	0.9976	0.0151	0.0002	3.1
	K	1.0633	-	0.0089	-	-	0.9973	0.0155	0.0003	3.3
	OK	1.0221	-	0.0101	-	-	0.9956	0.0203	0.0004	4.5
**Henderson and Pabis Model**										
CD 50	F	1.0237	-	0.0110	-	-	0.9942	0.0272	0.0008	6.3
	N	1.0242	-	0.0090	-	-	0.9968	0.0246	0.0005	4.4
	O	1.0020	-	0.0082	-	-	0.9989	0.0110	0.0001	2.3
	K	1.0204	-	0.0095	-	-	0.9945	0.0260	0.0008	5.6
	OK	1.0040	-	0.0106	-	-	0.9983	0.0142	0.0002	3.3
CD 60	F	1.0426	-	0.0118	-	-	0.9915	0.0326	0.0012	7.1
	N	1.0211	-	0.0107	-	-	0.9904	0.0338	0.0013	7.4
	O	1.0242	-	0.0090	-	-	0.9968	0.0205	0.0005	4.4
	K	1.0187	-	0.0101	-	-	0.9942	0.0257	0.0007	5.3
	OK	1.0252	-	0.0113	-	-	0.9911	0.0330	0.0012	7.4
CD 70	F	1.0135	-	0.0118	-	-	0.9941	0.0245	0.0007	5.1
	N	1.0026	-	0.0111	-	-	0.9964	0.0197	0.0004	4.3
	O	1.0291	-	0.0120	-	-	0.9947	0.0238	0.0006	4.9
	K	1.0095	-	0.0118	-	-	0.9965	0.0184	0.0004	3.9
	OK	0.9937	-	0.0111	-	-	0.9958	0.0205	0.0005	4.5
**Logistical Model**										
CD 50	F	0.8217	1.7679	0.0164	-		0.9986	0.0124	0.0001	2.9
	N	1.1789	2.1448	0.0123	-		0.9996	0.0068	0.0000	1.4
	O	3.9942	4.9340	0.0093	-		0.9993	0.0085	0.0000	1.8
	K	0.9293	1.8766	0.0138	-		0.9985	0.0128	0.0001	2.7
	OK	2.4868	3.4224	0.0126	-		0.9990	0.0100	0.0001	2.4
CD 60	F	0.5939	1.5568	0.0191	-		0.9992	0.0092	0.0000	2
	N	0.7680	1.7039	0.0162	-		0.9950	0.0226	0.0008	5.8
	O	1.1789	2.1448	0.0123	-		0.9996	0.0067	0.0000	1.4
	K	1.0250	1.9725	0.0142	-		0.9981	0.0141	0.0002	2.9
	OK	0.7368	1.6792	0.0172	-		0.9964	0.0197	0.0004	4.5
CD 70	F	1.3396	2.2843	0.0158	-		0.9969	0.0170	0.0004	3.6
	N	2.0664	2.9931	0.0136	-		0.9975	0.0154	0.0003	3.4
	O	1.3208	2.2983	0.0016	-		0.9990	0.0092	0.0000	1.9
	K	1.9187	2.8664	0.0147	-		0.9981	0.0130	0.0002	2.7
	OK	2.2933	3.1940	0.0134	-		0.9967	0.0173	0.0003	3.8
**Two-term Model**										
CD 50	F	0.3334	0.6903	0.0110	0.0110	-	0.9942	0.0272	0.0008	6.3
	N	0.3430	0.6812	0.0090	0.0090	-	0.9968	0.0205	0.0005	4.4
	O	0.0072	0.6648	0.0082	0.0082	-	0.9989	0.0110	0.0001	2.3
	K	0.3307	0.6896	0.0095	0.0095	-	0.9945	0.0260	0.0008	5.6
	OK	0.3254	0.6786	0.0106	0.0106	-	0.9983	0.0114	0.0002	3.3
CD 60	F	0.3336	0.7090	0.0118	0.0118	-	0.9915	0.0326	0.0013	7.1
	N	0.3286	0.6926	0.0108	0.0108	-	0.9891	0.0350	0.0014	7.4
	O	0.3430	0.6812	0.0090	0.0090	-	0.9968	0.0205	0.0005	4.4
	K	0.3290	0.6896	0.0101	0.0101	-	0.9942	0.0275	0.0008	5.3
	OK	0.3291	0.6961	0.0113	0.0113	-	0.9911	0.0330	0.0013	7.4
CD 70	F	0.3279	0.6856	0.0118	0.0118	-	0.9941	0.0245	0.0007	5.1
	N	0.3404	0.6622	0.0111	0.0111	-	0.9964	0.0197	0.0005	4.3
	O	0.3301	0.6990	0.0120	0.0120	-	0.9947	0.0238	0.0007	4.9
	K	0.3274	0.6822	0.0118	0.0118	-	0.9965	0.0184	0.0004	3.9
	OK	0.3233	0.6704	0.0111	0.0111	-	0.9958	0.0205	0.0005	4.5
**Newton Model**										
CD 50	F	-	-	0.0107	-	-	0.9952	0.0288	0.0009	6.6
	N	-	-	0.0087	-	-	0.9978	0.0228	0.0006	4.9
	O	-	-	0.0082	-	-	0.9990	0.0110	0.0001	2.3
	K	-	-	0.0093	-	-	0.9955	0.0273	0.0008	5.8
	OK	-	-	0.0105	-	-	0.9984	0.0143	0.0002	3.4
CD 60	F	-	-	0.0112	-	-	0.9937	0.0368	0.0015	8.1
	S	-	-	0.0104	-	-	0.9918	0.0349	0.0014	7.6
	O	-	-	0.0870	-	-	0.9978	0.0223	0.0006	4.9
	K	-	-	0.0098	-	-	0.9953	0.0269	0.0008	5.5
	OK	-	-	0.0109	-	-	0.9924	0.0345	0.0013	7.8
CD 70	F	-	-	0.0116	-	-	0.9948	0.0251	0.0007	5.3
	N	-	-	0.0111	-	-	0.9965	0.0020	0.0004	4.3
	O	-	-	0.0115	-	-	0.9962	0.0266	0.0008	5.5
	K	-	-	0.0117	-	-	0.9970	0.0189	0.0004	3.9
	OK	-	-	0.0112	-	-	0.9956	0.0206	0.0005	4.5
**Midilli et al., Model**										
CD 50	F	0.9615	0	0.0037	-	1.2267	0.9978	0.0155	0.0002	3.6
	N	0.9777	0	0.004	-	1.1627	0.9992	0.0092	0.0000	2
	O	0.9761	0	0.0053	-	1.0867	0.9991	0.0095	0.0001	2
	K	0.9641	0	0.0035	-	1.2046	0.9978	0.0154	0.0003	3.3
	OK	0.9787	0	0.0071	-	1.0822	0.9988	0.0117	0.0001	2.6
CD 60	F	0.9641	0	0.0028	-	1.3025	0.9985	0.0128	0.0002	2.8
	N	0.9555	0	0.0034	-	1.2344	0.9946	0.024	0.0007	5.2
	O	0.9778	0	0.0040	-	1.1627	0.9993	0.0093	0.0000	2
	K	0.9662	0	0.0040	-	1.1891	0.9973	0.0165	0.0003	3.4
	OK	0.9539	0	0.0031	-	1.2725	0.9952	0.0225	0.0006	5.1
CD 70	F	0.9707	0	0.0058	-	1.1491	0.9962	0.0187	0.0004	3.9
	N	0.9739	0	0.0070	-	1.0944	0.9971	0.0168	0.0003	3.7
	O	0.9787	0	0.0051	-	1.1794	0.9980	0.0137	0.0002	2.8
	K	0.9519	0	0.0044	-	1.2085	0.9964	0.0173	0.0004	3.6
	OK	0.9231	0	0.0030	-	1.2758	0.9927	0.0255	0.0255	5.6

CD: convective drying (50, 60, and 70 °C), VD: vacuum drying, FD: freeze drying, F: courgette without pretreatment, N: 3% NaCl solution, O: onion juice + 3% NaCl, K: kale juice + 3% NaCl, and OK: onion and kale juice (50:50) + 3% NaCl.

**Table 4:** Bioactive components: quercetin, total chlorophylls, total carotenoids, lutein, and zeaxanthin in courgette dried using different drying methods: convective drying by temperatures of 50 (CD 50), 60 (CD 60), and 70 °C (CD 70); vacuum drying (VD); and freeze drying (FD) when fresh (F), impregnated salt solution (N), onion juice (O), kale juice (K), or onion + kale juice (OK). Means within one column with a different letter superscript are significantly different homogeneous groups (*p* < 0.05).

Drying Method	Material	Chemical Composition (μg g d.m.^−1^)			
		Quercetin	Total Carotenoids	Total Chlorophylls	Lutein and Zeaxanthin
**Not dried**	F	1.82 ± 0.04 ^a^	298.87 ± 16.01 ^a^	59.21 ± 4.61 ^a^	184.91 ± 8.26 ^a^
	N	ND	279.44 ± 9.48 ^a^	39.02 ± 5.76 ^b^	166.42 ± 9.11 ^a^
	O	51.00 ± 2.63 ^b^	290.99 ± 18.33 ^a^	52.07 ± 5.25 ^a^	188.00 ± 10.87 ^a^
	K	1.90 ± 0.30 ^a^	349.49 ± 3.51 ^b^	89.83 ± 7.24 ^d^	218.44 ± 16.23 ^b^
	OK	11.00 ± 0.39 ^c^	329.99 ± 16.95 ^b^	83.27 ± 7.74 ^d^	203.11 ± 10.00 ^b^
CD 50	F	ND	28.36 ± 6.11 ^c^	11.30 ± 3.36 ^e^	12.54 ± 0.74 ^g^
	N	ND	103.73 ± 12.74 ^d^	25.81 ± 3.64 ^c^	52.60 ± 12.32 ^f^
	O	0.82 ± 0.22 ^d^	110.80 ± 20.61 ^d^	15.49 ± 2.70 ^e^	53.49 ± 5.50 ^f^
	K	ND	140.19 ± 25.14 ^d,e^	63.49 ± 12.23 ^a^	115.54 ± 20.18 ^d^
	OK	2.97 ± 0.72 ^a^	97.65 ± 11.76 ^d^	27.24 ± 2.77 ^c^	90.20 ± 9.66 ^e^
CD 60	F	ND	194.96 ± 34.52 ^e^	29.56 ± 5.86 ^c^	165.32 ± 18.86 ^a^
	N	ND	132.61 ± 12.61 ^d,e^	18.24 ± 1.77 ^e^	100.80 ± 6.94 ^d^
	O	20.38 ± 1.95 ^e^	124.38 ± 28.91 ^d,e^	26.55 ± 4.65 ^c^	135.06 ± 15.60 ^c^
	K	ND	346.85 ± 7.65 ^d,e^	85.96 ± 18.97 ^d^	201.82 ± 25.56 ^b^
	OK	8.04 ± 2.37 ^c^	184.00 ± 16.46 ^e^	31.68 ± 3.86 ^b^	123.95 ± 5.64 ^c^
CD 70	F	ND	186.76 ± 36.73 ^e^	28.51 ± 6.31 ^c^	125.49 ± 17.90 ^c^
	N	ND	214.09 ± 14.23 ^f^	30.72 ± 2.91 ^b^	157.58 ± 6.28 ^c^
	O	50.94 ± 9.00 ^b^	152.83 ± 20.46 ^e^	21.88 ± 3.29 ^c^	135.31 ± 3.86 ^c^
	K	1.85 ± 0.10 ^a^	289.35 ± 27.42 ^a^	67.34 ± 11.59 ^a^	216.42 ± 25.14 ^b^
	OK	5.58 ± 1.05 ^a,c^	157.17 ± 4.05 ^e^	24.12 ± 0.57 ^c^	129.17 ± 2.93 ^c^
VD	F	0.75 ± 0.08 ^d^	174.10 ± 13.63 ^e,f^	25.84 ± 3.05 ^c^	138.26 ± 5.81 ^c^
	N	ND	134.15 ± 13.54 ^d,f^	29.93 ± 3.97 ^c^	105.63 ± 14.12 ^d^
	O	11.80 ± 0.80 ^c^	151.82 ± 6.11 ^e^	23.81 ± 0.36 ^c^	116.69 ± 6.23 ^d^
	K	ND	161.99 ± 21.90 ^e^	33.18 ± 2.35 ^b^	140.61 ± 22.64 ^c^
	OK	10.54 ± 0.50 ^c^	266.71 ± 28.81 ^a^	52.27 ± 6.86 ^a^	177.99 ± 22.67 ^a^
FD	F	ND	266.55 ± 48.67 ^a^	53.84 ± 9.97 ^a^	180.94 ± 18.50 ^a^
	N	ND	158.66 ± 45.13 ^e^	34.46 ± 11.12 ^b^	134.63 ± 39.24 ^c^
	O	41.84 ± 7.22 ^b^	276.04 ± 43.72 ^a^	50.43 ± 12.00 ^a^	162.69 ± 6.32 ^a^
	K	0.86 ± 0.51 ^d^	267.38 ± 12.86 ^a^	75.34 ± 4.69 ^d^	185.89 ± 6.39 ^a^
	OK	7.44 ± 1.50 ^a,c^	248.78 ± 18.48 ^a^	48.53 ± 3.76 ^a^	161.32 ± 12.68 ^a^

**Table 5:** Impregnating solutions.

Symbol	Infiltration Solutions	°Bx
N	3% sodium chloride solution (NaCl)	4.2
K	Freshly squeezed kale juice + 3% NaCl	11.5
O	Freshly squeezed onion juice + 3% NaCl	10.5
KO	Freshly squeezed onion juice and kale juice (50:50) + 2% NaCl	9.5

**Table 6:** Mathematical models used to describe the kinetics of the courgette drying process.

Numer	Model Name	Equation	Source
1	Page	$MR=exp\left(-k\cdot \tau^{a}\right)$	[47]
2	Henderson and Pabis	$MR=a\cdot exp\left(-k\tau \right)$	[48]
3	Newton	$MR=exp\left(-k\tau \right)$	[49]
4	Midilli et al.	$MR=a\cdot exp\left(-k\cdot \tau^{c}\right)+b\cdot \tau$	[50]
5	Two-term	$MR=a\cdot exp\left(-k\tau \right)+b\cdot exp\left(-k_{i}\cdot \tau \right)$	[51]
6	Logistical	$MR=\frac{b}{\left(1+a\cdot \exp\left(k\cdot \tau \right)\right)}$	[52]

## Data Availability

Additional data are available on request to the authors.

## Sample Availability

Samples of the compounds are available from the authors.
